# Complete Chloroplast Genome Sequence and Phylogenetic Analysis of *Quercus acutissima*

**DOI:** 10.3390/ijms19082443

**Published:** 2018-08-18

**Authors:** Xuan Li, Yongfu Li, Mingyue Zang, Mingzhi Li, Yanming Fang

**Affiliations:** 1 Co-Innovation Center for Sustainable Forestry in Southern China, College of Biology and the Environment, Key Laboratory of State Forestry Administration on Subtropical Forest Biodiversity Conservation, Nanjing Forestry University, 159 Longpan Road, Nanjing 210037, China; xuanli18851128817@163.com (X.L.); liyongfu199417@gmail.com (Y.L.); sanskritm@163.com (M.Z.); 2 Genepioneer Biotechnologies Co. Ltd., Nanjing 210014, China; limzhi87@foxmail.com

**Keywords:** *Quercus*, chloroplast genome, phylogenetic relationship

## Abstract

*Quercus acutissima*, an important endemic and ecological plant of the *Quercus* genus, is widely distributed throughout China. However, there have been few studies on its chloroplast genome. In this study, the complete chloroplast (cp) genome of *Q. acutissima* was sequenced, analyzed, and compared to four species in the Fagaceae family. The size of the *Q. acutissima* chloroplast genome is 161,124 bp, including one large single copy (LSC) region of 90,423 bp and one small single copy (SSC) region of 19,068 bp, separated by two inverted repeat (IR) regions of 51,632 bp. The GC content of the whole genome is 36.08%, while those of LSC, SSC, and IR are 34.62%, 30.84%, and 42.78%, respectively. The *Q. acutissima* chloroplast genome encodes 136 genes, including 88 protein-coding genes, four ribosomal RNA genes, and 40 transfer RNA genes. In the repeat structure analysis, 31 forward and 22 inverted long repeats and 65 simple-sequence repeat loci were detected in the *Q. acutissima* cp genome. The existence of abundant simple-sequence repeat loci in the genome suggests the potential for future population genetic work. The genome comparison revealed that the LSC region is more divergent than the SSC and IR regions, and there is higher divergence in noncoding regions than in coding regions. The phylogenetic relationships of 25 species inferred that members of the *Quercus* genus do not form a clade and that *Q. acutissima* is closely related to *Q. variabilis*. This study identified the unique characteristics of the *Q. acutissima* cp genome, which will provide a theoretical basis for species identification and biological research.

## 1. Introduction

Oak trees provide humans with materials used in food, clothing, and houses, while oak forests supply living organisms and animals with comfortable habitats, good air, and sufficient and pure moisture. Oak trees are linked to Chinese culture, and are also often called eucalyptus or pecking trees. In China, eucalyptus is regarded as a mysterious tree, growing silently, watching its ancestors forge ahead, and passing through generation to generation. Many countries regard oaks as sacred trees, and consider them to be magical and a symbol of longevity, strength, and pride.

The genus *Quercus* L. (Oak) contains more than 400 species that are widespread in the northern hemisphere [1]. These species play important roles in China’s forest ecosystem. *Quercus* L. (Oak)’s taxonomy, genetic structure, and breeding is complicated because of its wide variety of species, diverse forms, complex habitat conditions, and gene exchanges between species. Many studies have used nuclear simple sequence repeat (SSR) chloroplast DNA makers to study phylogeny and population variation [2,3]. Previously, studies found a conflict (inconsistency) between the phylogeny of plastid data and nuclear data in Senecioneae and Neotropical Catasetinae [4,5]. Therefore, it is not sufficient to study *Quercus* simply by using plastid regions. With the rapid development of next-generation sequencing, genome acquisition is now cheaper and faster than traditional Sanger sequencing. Complete chloroplast (cp) genome size data will be necessarily used to infer the phylogenetic relationship of *Quercus* or Fagaceae in future studies.

The genus is characterized by a high variability of morphological and ecological traits, the occurrence of mixed stands, the presence of large population sizes, and high levels of gene flow within the *Quercus* complex [6,7,8,9,10,11]. A new classification of *Quercus* L. was proposed by Denk with eight sections: *Cyclobalanopsis*, *Cerris*, *Ilex*, *Lobatae*, *Quercus*, *Ponticae*, *Protobalanus*, and *Virentes* [12]. In China, *Quercus* is divided into five morphology-based sections: *Quercus*, *Aegilops*, *Heterobalanus*, *Engleriana*, and *Echinolepides* [13,14,15]. Due to incomplete sampling and the use of markers with insufficient phylogenetic signals and complex evolutionary problems, the relationships among *Quercus* species are not fully understood.

*Q. acutissima* is an ecological and economic tree species in deciduous broad-leaved forests in the temperate zone of East Asia, widely distributed on the Hu Huanyong line or in Southeast China (latitude from 18° to 41° N and longitude from 91° to 123° E) [16]. This line from Heilongjiang Province to Tengchong, Yunnan Province, is roughly inclined in a 45° straight line. The development, origin, and reproduction of China are linked with *Q. acutissima*. Therefore, we need to protect, cultivate, and utilize *Q. acutissima*, and this has received substantial attention in phylogeny and biogeography studies. Most previous studies have focused on its population structure [17], breeding [18], forest management [19], and physiology [20]. Studies on the genetic variation of *Q. acutissima* using simple sequence repeat (SSR) and cpDNA makers have been carried out in China and South Korea [16,21]. According to this research, the distribution of *Q. acutissima* often overlaps with other oak trees, i.e., *Q. variabilis* and *Q. chenii* [22]. There is often a variety of species found in the population, although this has usually been determined from a comparison of morphology, rather than at a molecular level. Therefore, an analysis of the complete cp genome of *Q. acutissima* will help to identify the species further.

In the present study, we constructed the whole chloroplast genome of *Q. acutissima* by using next-generation sequencing and applying a combination of de novo and reference-guided assembly. Here, we describe the whole chloroplast genome sequence of *Q. acutissima* and the characterization of long repeats and simple sequence repeats (SSRs). We compare and analyze the chloroplast genome of *Q. acutissima* and the chloroplast genome of other members of Fagaceae. It is expected that the results will provide a theoretical basis for the determination of phylogenetic status and future scientific research.

## 2. Results and Discussion

### 2.1. Features of Q. Acutissima cpDNA

**Figure 1:**
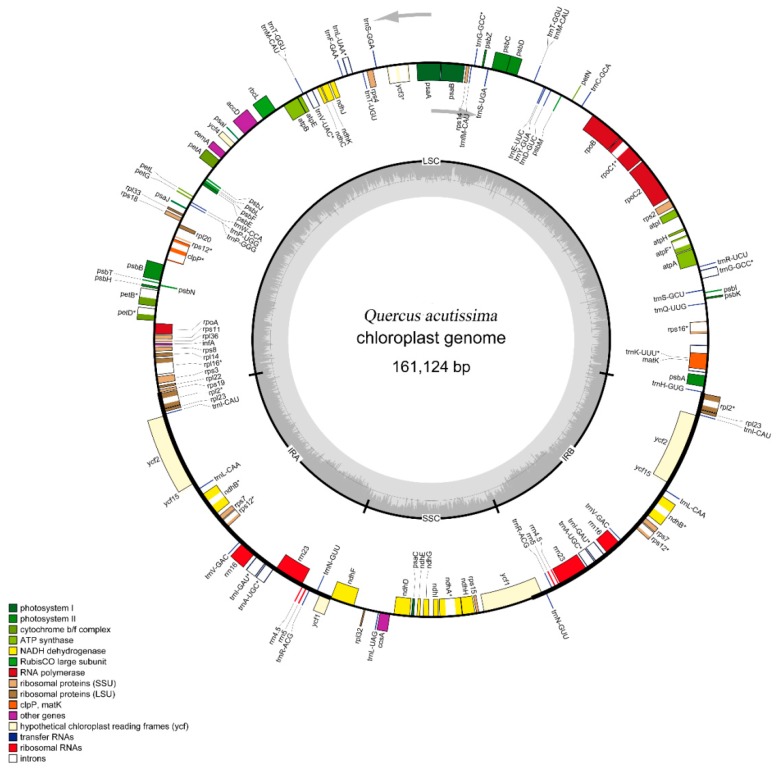
Chloroplast genome map of *Q. acutissima*. Genes inside the circle are transcribed clockwise, and those outside are transcribed counterclockwise. Genes of different functions are color-coded. The darker gray in the inner circle shows the GC content, while the lighter gray shows the AT content.

**Figure A1:**
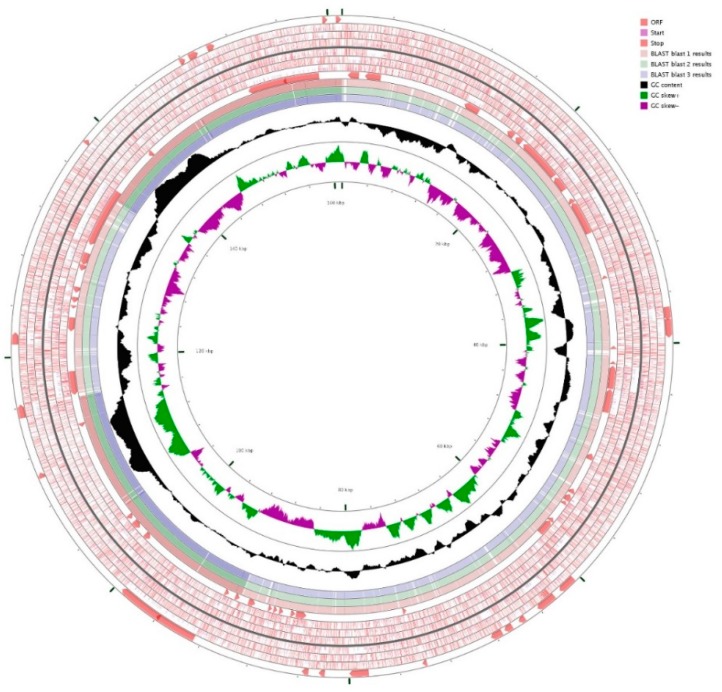
BLAST result of the chloroplast genome and the GC stew of *Q. acutissima.* BlAST 1 represents *L. balansae*; BlAST 2 represents *Q. variabilis*; BlAST 3 represents *Q. dolicholepis*.

A total number of 63 million pair-end reads were produced with 9.82 Gb of clean data. Data from all of the reads were deposited in the NCBI Sequence Read Archive (SRA) under accession number MH607377. The size of the complete cp genome is 161,124 bp (Figure 1). The cp genome displayed a typical quadripartite structure, including a pair of IR (25,816 bp) separated by the large single copy (LSC; 90,423 bp) and small single copy (SSC; 19,069 bp) regions (Figure 1 and Table 1). The DNA G + C contents of the LSC, SSC, and IR regions, and the whole genome are 34.62, 30.84, 42.78, and 36.08 mol %, respectively, which is also similar to the chloroplast genomes of other *Quercus* species (Figure A1; Table 2). The DNA G + C content is a very important indicator of species affinity [23]. It is obvious that the DNA G + C content of the IR region is higher than that of other regions (LSC, SSC). This phenomenon is very common in other plants [23,24]. GC skewness has been shown to be an indicator of DNA lead chains, lag chains, replication origin, and replication terminals [25,26,27].

Plant chloroplast genomes may have 63–209 genes, but most are concentrated between 110 and 130, with a highly conserved composition and arrangement, including photosynthetic genes, chloroplast transcriptional expression-related genes, and some other protein-coding genes [28]. In the *Q. acutissima* chloroplast genome, 136 functional genes were predicted and divided into six groups, including eight rRNA genes, 40 tRNA genes, and 88 protein-coding genes (Table 1 and Table 3). In addition, 14 tRNA genes, eight rRNA genes, and 15 protein-coding genes are duplicated in the IR regions (Figure 1). The LSC region includes 62 protein-coding and 25 tRNA genes, while the SSC region includes 13 protein-coding genes (Table A1). 

Based on the protein-coding sequences and tRNA genes, the frequency of codon usage was estimated for the *Q. acutissima* cp genome and is summarized in Table A2. In total, all genes are encoded by 6311 codons. Among these, leucine, with 2824 (44.4%) codons, is the most frequent amino acid in the cp genome, and cysteine, with 293 (1.1%), is the least frequent (Table 3). A- and U-ending codons are common. The most preferred synonymous codons (relative synonymous codon usage values (RSCU) > 1) end with A or U [23,29].

In total, we found 23 intron-containing genes, including 15 protein-coding genes, and eight tRNA genes (Table 4). 21 genes (13 protein-coding and eight tRNA genes) contain one intron, and two genes (*ycf3* and *clpP*) contain two introns. The *trnK-UUU* has the largest intron (2505 bp), and the *trnL-UAA* has the smallest intron (483bp). Studies have shown that *ycf3* is required for stable accumulation of photosystem I complexes [30]. Therefore, we speculate that the *ycf3* intron gain of *Q. acutissima* may be helpful for further study of the mechanism of photosynthesis evolution.

### 2.2. Comparative Analysis of Genomic Structure

**Figure 2:**
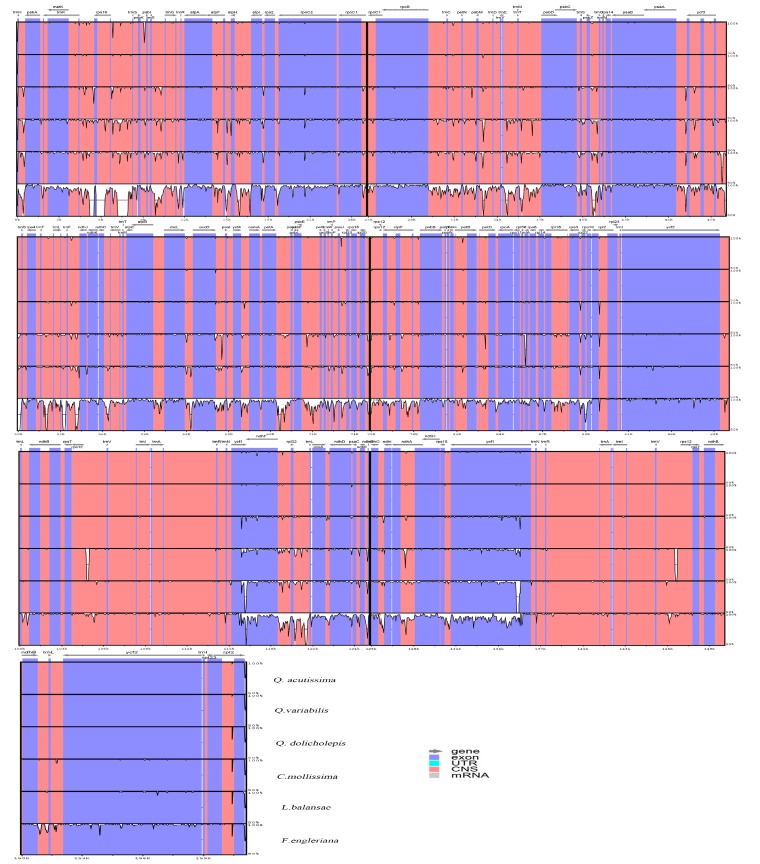
Complete chloroplast genome comparison of six species using the chloroplast genome of *Q. variabilis* as a reference. The grey arrows and thick black lines above the alignment indicate the genes’ orientations. The Y-axis represents the identity from 50% to 100%.

**Figure A2:**
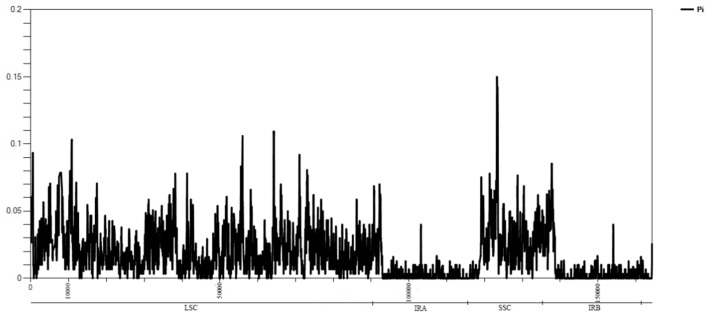
Percentage of variation in the complete cp genomes of the six species. The regions are oriented according to their locations in the genome.

The chloroplast sequence are often used to measure the genetic diversity within a species, the gene flow between species, and the size of ancestral populations of separated sister species [31]. Thus, it is necessary to understand the chloroplast differences between species. The complete cp genome sequence of *Q. acutissima* was compared to those of *Q. variabilis*, *Q. dolicholepis*, *Castanea mollissima*, *Lithocarpus balansae*, and *Fagus engleriana. F. engleriana* has the smallest cp genome with the largest IR region (51,784 bp), and *Q. dolicholepis* has the largest cp genome (Table 1). We assumed that the different lengths of the SSC and IR regions is the main reason for variety in sequence lengths. To verify the possibility of genome divergence, sequence identity was calculated for six species’ chloroplast DNA using the program mVISTA with *Q. variabilis* as a reference (Figure 2). The results of this comparison revealed that LSC regions are more divergent than SSC and IR regions and that higher divergence is found in noncoding than in coding regions. The complete cp genome sequence of *F. engleriana* is quite different from the five other plants. There was no significant difference between the chloroplast genome sequences of evergreen and deciduous trees. At the same time, the results of the sliding window indicated that the location of the variation in the cp genome among the six species occurred in the LSC and SSC regions (Figure A2). Significant variation was found in coding regions of some genes, including *psbI*, *rpl33*, *petB*, *rpl2*, *rps16*, *rpoC2*, *ndhK*, *ycf2*, *ycf1*, and *ndhI*. The highest divergence in noncoding regions was found in the intergenic regions of *trnK-rps16*, *rps 16-trnQ*, *psbK-psbI*, *trnS-trnG*, *atpH-atpI*, *atpI-rps2*, *rpoB-trnC*, *trnC-petN*, *psbM*-*trnD*, *trnD-trnY*, *trnE-trnM*, *trnT-petD*, *psbZ-trnG*, *trnT-trnL*, *trnF-ndhJ*, *rbcL*-*accD*, *psaI*-*ycf4*, *ycf4-cemA*, *petA-psbL*, *psaJ*-*rpl33*, *clpP*-*psbB*, *rpl14-rpl16, ndhF-rpl32, ccsA-ndhD, ndhD-psaC*, and *rps15-ycf1*.

**Figure 3:**
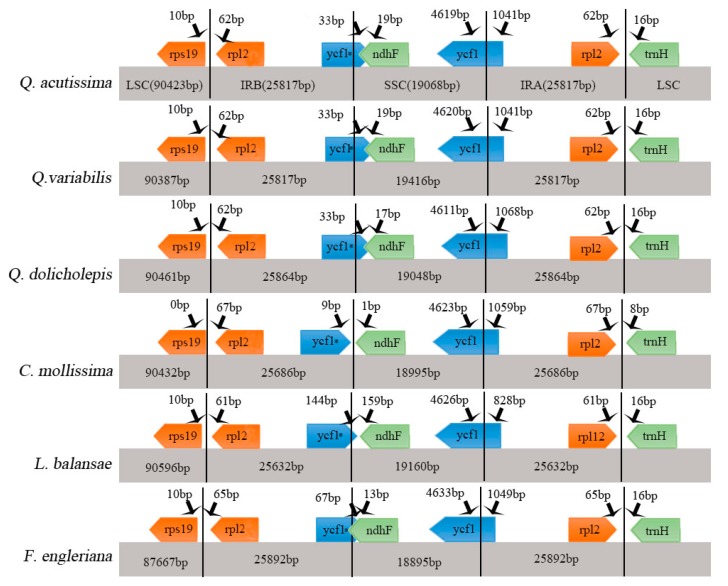
Comparison of the large single copy (LSC), small single copy (SSC), and inverted repeat (IR) regions in chloroplast genomes of four species. Genes are denoted by colored boxes. The gaps between the genes and the boundaries are indicated by the base lengths (bp). Extensions of the genes are indicated above the boxes.

The contraction and expansion of the IR region at the borders play important roles in evolution. They are common evolutionary events and a major cause of changes in the size of the chloroplast genome. They may also cause variation in the length of angiosperm plastid genome [32,33,34]. Detailed comparisons of the IR–SSC and IR–LSC boundaries among the cp genomes of the above six Fagaceae species were presented in Figure 3. The IR regions are relatively highly conserved in the *Quercus* genus—the *rpl2* gene in the *Quercus* cp genome is shifted by 62 bp from IRb to LSC at the LSC/IRb border, and by 62 bp from IRa to LSC at the IRa/LSC border. Compared to other species in the genus, the range of the IRa/SSC regions changes greatly. Compared with evergreen and deciduous species, we found significant differences in IRb/SSC. Some reports showed that *ycf1* is necessary for plant viability and encodes *Tic214*, an important component of the *Arabidopsis TIC* complex [35,36]. The *ycf1* gene crossed the SSC/IRb region, with 1041bp of *ycf1_like* within IRb (incompletely duplicated in IRb). The SSC/IRa junction is located in the *ycf1* region in all Fagaceae species chloroplast genomes and extends into the SSC region by different lengths depending on the genome *(Q. acutissima*, 4619 bp; *Q. variabilis*, 4620 bp; *Q. dolicholepis,* 4611 bp; *C. mollissima*, 4623 bp; *L. balansae*, 4626 bp; *F. engleriana*, 4633 bp); the IRa region includes 1041, 1041, 1068, 1059, 828, and 1049 bp of the *ycf1* gene. 

### 2.3. Long-Repeat and SSR Analysis

For the repeat structure analysis (Table 5), 31 forward and 22 inverted repeats were detected in the *Q. acutissima* cp genome. Most of these repeats are between 19 and 46 bp. The longest forward repeat is 46 bp in length and is located in the LSC region. A total of 35, 18, and eight repeats were found in the LSC, SSC, IR regions, respectively. Seven forward repeats were located in IR, including one repeat associated with *ycf1* genes and one repeat related to the *trnV-UAC* and *trnA-UGC* genes. Most repeats in the intergenic spacers are distributed in the LSC region. Ten repeats are distributed in the SSC region, and only four of them are in the intergenic spacers. 

As chloroplast-specific SSRs are uniparentally inherited and are inclined to undergo slipped-strand mispairing, they are often used in population genetics, species identification, and evolutionary process research of wild plants [37,38]. In addition, chloroplast genome sequences are highly conserved, and the SSR primer for chloroplast genomes can be transferred across species and genera. Yoko et al. used six maternally inherited chloroplast (cpDNA) simple sequence repeat (SSR) markers to study the genetic variation in *Q. acutissima* [39]. In this study, a total of 65 SSRs were found in *Q. acutissima*, most of them distributed in LSC and SSC and partly distributed in IR. These included 61 mononucleotide SSRs (93.85%) and four dinucleotide SSRs (6.15%) (Table 6). Compared with other *Quercus* species, fewer types of SSRs were identified in *Q. acutissima* [40]. Among them, two SSRs belonged to the C type, and the others all belonged to the A/T types. These results are consistent with the hypothesis that cpSSRs are generally composed of short polyadenine (polyA) or polythymine (polyT) repeats and rarely contain tandem guanine (G) or cytosine (C) repeats [41]. We also found that 12 SSRs were located in genes, and the remaining were all located in intergenic regions. These cpSSR markers could be used to examine the genetic structure, diversity, differentiation, and maternity in *Q. acutissima* and its relative species in future studies.

### 2.4. Phylogenetic Analysis

**Figure 4:**
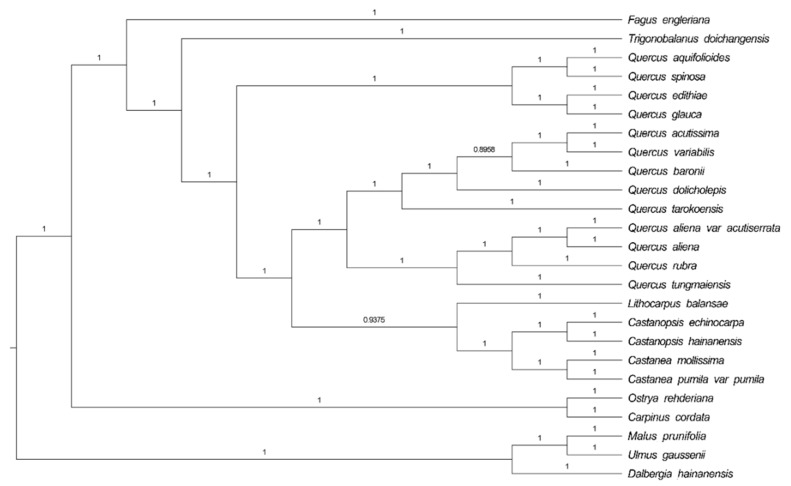
Bayesian inference (BI) phylogenetic tree reconstruction including 25 species based on all chloroplast genomes. *Malus prunifolia* and *Ulmus gaussenii* were used as the outgroup.

Phylogenetic analysis was completed on an alignment of concatenated nucleotide sequences of all chloroplast genomes from 25 angiosperm species (Figure 4). We used the Bayesian inference (BI) method based on RAxML to build a phylogenetic tree, and *Malus prunifolia* and *Ulmus gaussenii* were used as the outgroup. Support is generally high for almost all relationships inferred from all chloroplast genome data based on BI methods (the support values have a range of 0.8956 to 1). It is noteworthy that the species in genus *Quercus* do not form a clade. Several evergreen tree species gather together to form one clade. *Q. acutissima* and *Q. variabilis* are sister species and are frequently mixed in Chinese endemic species; the second clade splits into two subclades. *F. engleriana* is in the top position, while *Q. acutissima* appears to be more closely related to *Q. variabilis*, *Q. dolicholepis*, and *Q. baronii*. In general, the topologies of the other branches (genus *Fagus*, *Trigonobalanus*, *Lithocarpus*, and *Castanopsis*) are almost the same based on two nuclear loci (ITS and CRC) [3]. 

## 3. Materials and Methods

### 3.1. Sampling, DNA Extraction, Sequencing, and Assembly

*Q. acutissima* was planted in Nanjing Forestry University and Zijin Mountain in Nanjing, China (32°04′ N, 118°48′ E; 32°04′ N, 118°50′ E), respectively. Fresh leaves were collected and wrapped in ice and immediately stored at −80 °C until analysis. Genomic DNA was isolated by the modified method CTAB [42]. Agarose gel electrophoresis and one drop spectrophotometer (OD-1000, Shanghai Cytoeasy Biotech Co., Ltd., Shanghai, China) were used to detect DNA integrity and quality. Shotgun libraries (250 bp) were constructed using pure DNA according to the manufacturer’s instructions. Sequencing was performed with an Illumina Hiseq 2500 platform (Nanjing, China), yielding at least 9.82 GB of clean data for *Q. acutissima*. Firstly, all of the raw reads were trimmed by Fastqc. Next, we performed a BLAST analysis between trimmed reads and references (*Q. variabilis* and *Q. dolicholepis*) to extract cp-like reads. Finally, we used the chloroplast-like reads to assemble sequences using NOVOPlasty [43]. NOVOPlasty assembled part reads and stretched as far as possible until a circular genome formed. When the assembly result was within the expected range, the overlap was larger than 200 bp, and the assembly formed a ring.

### 3.2. Annotation and Analysis of the cpDNA Sequences

CpGAVAS was used to annotate the sequences; DOGMA (http://dogma.ccbb.utexas.edu/) and BLAST were used to check the results of the annotation [44,45]. tRNAscanSE was used to identify the tRNAs [46]. The circular gene maps of the species of *Q. acutissima* were drawn using the OGDRAWv1.2 program [47] (http://ogdraw.mpimp-golm.mpg.de/). An analysis of variation in synonymous codon usage, relative synonymous codon usage values (RSCU), codon usage, and the GC content of the complete plastid genomes and commonly analyzed CDS was conducted. MISA(available online: http://pgrc.ipk-gatersleben.de/misa/misa.html) [48] and REPuter (available online: https://bibiserv.cebitec.uni-bielefeld.de/reputer/) [49] was used to visualize the SSRs and long repeats, respectively.

### 3.3. Genome Comparison

MUMmer [50] was used for pairing sequence alignment of the cp genome. The mVISTA [51] program was applied to compare the complete cp genome of *Q. acutissima* to the other published cp genomes of its related species, i.e., *Q. variabilis* (KU240009), *Q. dolicholepis* (KU240010), *C. mollissima* (HQ336406), *L. balansae* (KP299291), and *F. engleriana* (KX852398) with the shuffle-LAGAN mode [52], using the annotation of *Q. variabilis* as a reference. 

### 3.4. Phylogenetic Analysis

Phylogenies were constructed by Bayesian inference (BI) analysis using the 25 cp genome of the Fagaceae species sequences from the NCBI Organelle Genome and Nucleotide Resources database. The sequences were initially aligned using MAFFT [53]. Then, the visualization and manual adjustment of multiple sequence alignment were conducted in BioEdit [54]. An IQ-tree was used to select the best-fitting evaluation of models of nucleotide sequences [55]. TVM + F + R4 and GTR + G were selected as the best substitution models for the BI analyses. BI analyses were conducted using Mrbayes [56]. *Malus prunifolia* (NC_031163), and the *Ulmus gaussenii* (NC_037840) were used as the outgroups.

## 4. Conclusions

In this study, we reported and analyzed the complete cp genome of *Q. acutissima*, an endemic and ecological tree species in China. The chloroplast genome was shown to be more conservative with similar characteristics to other genus *Quercus* species. Compared to the cp genomes of five other oak species, its LSC were shown to be more divergent among the four regions, and noncoding regions showed higher divergence. An analysis of the phylogenetic relationships among six species found *Q. acutissima* to be closely related to *Q. variabilis*. The developmental position of the tree in the Fagaceae family is consistent with previous studies. The results of this study provide an assembly of a whole chloroplast genome of *Q. acutissima* which might facilitate genetics, breeding, and biological discoveries in the future.

## Figures and Tables

**Table 1:** Summary of five *Quercus* chloroplast genome features.

Genome Features	*Q. acutissima*	*Q. variabilis*	*Q. dolicholepis*	*C. mollissima*	*L. balansae*	*F. engleriana*
Genome size (bp)	161,124	161,077	161,237	160,799	161,020	158,346
LSC length (bp)	90,423	90,387	90,461	90,432	90,596	87,667
SSC length (bp)	19,068	19,056	19,048	18,995	19,160	18,895
IR length (bp)	51,632	51,634	51,728	51,372	51,264	51,784
Number of genes	136	134	134	130	134	131
Number of protein–coding genes	88	86	86	83	87	83
Number of tRNA genes	40	40	40	37	39	40
Number of rRNA genes	8	8	8	8	8	8

**Table 2:** Base composition of the *Q. acutissima* chloroplast genome.

Region	A (%)	T (U) (%)	C (%)	G (%)	A + T (%)	G + C (%)
LSC	31.99	33.4	17.74	16.88	65.39	34.62
SSC	34.46	34.71	16.24	14.6	69.17	30.84
IR	28.61	28.61	21.39	21.39	57.22	42.78
Total	31.69	32.24	18.46	17.62	63.93	36.08

**Table 3:** List of genes annotated in the cp genomes of *Q. acutissima* sequenced in this study.

Function	Genes
RNAs, transfer	*trnH-GUG*, *trnK-UUU*, *trnQ-UUG*, *trnS-GCU*, *trnG-GCC*, *trnR-UCU*, *trnC-GCA*, *trnD-GUC*, *trnY-GUA*, *trnE-UUC*, *trnT-GGU*, *trnM-CAU*, *trnS-UGA*, *trnG-GCC*, *trnfM-CAU*, *trnS-GGA*, *trnT-UGU*, *trnL-UAA*, *trnF-GAA*, *trnV-UAC*, *trnM-CAU*, *trnT-GGU*, *trnW-CCA*, *trnP-UGG*, *trnP-GGG*, trnI *-*CAU*, *trnL-CAA* *, *trnV-GAC*, trnI-GAU *, *trnA-UGC*, *trnR-ACG*, *trnN-GUU*, *trnL-UAG*, *trnN-GUU*, *trnR-ACG*, *trnA-UGC*, *trnV-GAC*
RNAs, ribosomal	rrn23 *, rrn16 *, rrn5 *, rrn4.5 *
Transcription and splicing	rpoC1 *, *rpoC2*, *rpoA*, *rpoB*
Translation, ribosomal proteins	
Small subunit	*rps2*, *rps3*, *rps4*, *rps7*, *rps8*, *rps11*, rps12 **, *rps14*, *rps15*, rps16 *, *rps18*, *rps19*
Large subunit	rpl2 *, *rpl14*, rpl16 *, *rpl20*, *rpl22*, *rpl23*, *rpl32*, *rpl33*, *rpl36*
Photosynthesis	
ATP synthase	*atpE*, *atpB*, *atpA*, atpF *, *atpH*, *atpI*
Photosystem I	*psaI*, *psaB*, *psaA*, *psaC*, *psaJ*, ycf3 *, *ycf4*
Photosystem II	*psbD*, *psbC*, *psbZ*, *psbT*, *psbH*, *psbK*, *psbI*, *psbJ*, *psbF*, *psbE*, *psbM*, *psbN*, *psbL*, *psbA*, *psbB*
Calvin cycle	*rbcL*
Cytochrome complex	*petN*, *petA*, *petL*, *petG*, petB *, petD *
NADH dehydrogenase	ndhB *, *ndhI*, *ndhK*, *ndhC*, *ndhF*, *ndhD*, *ndhG*, *ndhE*, *ndhA*, *ndhH*, *ndhJ*
Others	*inFA*, ycf15 *, ycf1 *, ycf2 *, *accD*, *cemA*, *ccsA*, clpP **

* Genes containing one intron; ** genes containing two introns.

**Table 4:** The lengths of exons and introns in genes with introns in the *Q. acutissima* chloroplast genome.

Gene	Location	Exon I (bp)	Intron I (bp)	Exon II (bp)	Intron II (bp)	Exon III (bp)
*rps16*	LSC	42	898	195		
*atpF*	LSC	144	780	411		
*rpoC1*	LSC	432	827	1626		
*ycf3*	LSC	127	718	228	778	155
*clpP*	LSC	69	844	294	649	228
*petB*	LSC	6	841	642		
*petD*	LSC	9	640	474		
*rpl16*	LSC	9	1102	399		
*rpl2*	RepeatA	390	628	471		
*ndhB*	RepeatA	777	680	756		
*rps12*	RepeatA	10	537	231		
*ndhA*	SSC	551	1040	541		
*rps12*	RepeatB			232	536	26
*ndhB*	RepeatB	777	680	756		
*rpl2*	RepeatB	390	628	471		
*trnG-GCC*	LSC	23	734	37		
*trnK-UUU*	LSC	37	2505	35		
*trnL-UAA*	LSC	35	483	50		
*trnV-UAC*	LSC	36	630	37		
*trnI-GAU*	RepeatA	42	950	35		
*trnA-UGC*	RepeatA	38	800	35		
*TRNA-UGC*	RepeatB	38	800	35		
*trnI-GAU*	RepeatB	42	950	35		

**Table 5:** Long repeat sequence in the *Q. acutissima* chloroplast genome.

ID	Repeat Start I	Type	Size (bp)	Repeat Start 2	Mismatch (bp)	E-Value	Gene	Region
1	6831	F	46	6853	0	1.47 × 10^−18^	IGS	LSC
2	11,847	R	31	11,847	0	1.58 × 10^−9^	IGS	LSC
3	6818	R	26	6818	0	1.62 × 10^−6^	*rps16*	LSC
4	47,242	F	25	47,264	0	6.49 × 10^−6^	IGS	LSC
5	6831	F	24	6875	0	2.59 × 10^−5^	IGS	LSC
6	115,801	F	24	135,722	0	2.59 × 10^−5^	*ycf1*	IRA; IRB
7	113,545	F	23	113,576	0	1.04 × 10^−4^	IGS	IRA
8	118,844	R	23	118,844	0	1.04 × 10^−4^	IGS	IRA
9	137,948	F	23	137,979	0	1.04 × 10^−4^	IGS	IRB
10	11,371	F	22	41,193	0	4.15 × 10^−4^	*trnG-GCC* (exon), *trnG-GCC*	LSC
11	9536	F	21	39,849	0	1.66 × 10^−3^	*trnS-UGA*, *trnS-GCU*	LSC
12	10,319	F	21	18,682	0	1.66 × 10^−3^	IGS	LSC
13	117,049	R	21	117,049	0	1.66 × 10^−3^	*ndhF*	SSC
14	36,478	F	20	53,719	0	6.64 × 10^−3^	IGS	LSC
15	53,720	F	20	130,481	0	6.64 × 10^−3^	IGS	LSC; SSC
16	55,907	R	20	55,907	0	6.64 × 10^−3^	*atpB*	LSC
17	57,271	F	20	142,064	0	6.64 × 10^−3^	*trnV-UAC*, *trnA-UGC*	LSC; IRB
18	105,331	F	20	105,349	0	6.64 × 10^−3^	IGS	IRA
19	146,178	F	20	146,196	0	6.64 × 10^−3^	IGS	IRB
20	4930	F	19	36,476	0	2.66 × 10^−2^	IGS	LSC
21	8915	R	19	8915	0	2.66 × 10^−2^	IGS	LSC
22	13,541	R	19	76,642	0	2.66 × 10^−2^	*atpA*	LSC
23	18,685	R	19	118,842	0	2.66 × 10^−2^	*clpP*	LSC; SSC
24	21,297	R	19	54,183	0	2.66 × 10^−2^	*rpoC2*	LSC
25	36,479	F	19	130,481	0	2.66 × 10^−2^	IGS	LSC; SSC
26	39,957	R	19	39,957	0	2.66 × 10^−2^	IGS	LSC
27	62,040	R	19	62,040	0	2.66 × 10^−2^	IGS	LSC
28	64,751	R	19	64,751	0	2.66 × 10^−2^	IGS	LSC
29	69,026	R	19	69,026	0	2.66 × 10^−2^	IGS	LSC
30	71,277	R	19	71,277	0	2.66 × 10^−2^	IGS	LSC
31	72,561	R	19	72,561	0	2.66 × 10^−2^	IGS	LSC
32	4430	R	18	4430	0	1.06 × 10^−1^	IGS	LSC
33	4437	F	18	24,828	0	1.06 × 10^−1^	*rpoC1* (intron)	SSC
34	4935	F	18	52,105	0	1.06 × 10^−1^	IGS	LSC
35	4938	F	18	118,695	0	1.06 × 10^−1^	IGS	LSC
36	6813	F	18	6847	0	1.06 × 10^−1^	IGS	LSC
37	6813	F	18	6869	0	1.06 × 10^−1^	IGS	LSC
38	6817	F	18	127,945	0	1.06 × 10^−1^	*ndhA* (intron)	LSC
39	7369	F	18	7387	0	1.06 × 10^−1^	IGS	LSC; SSC
40	7465	R	18	7465	0	1.06 × 10^−1^	IGS	LSC; SSC
41	8589	R	18	34,768	0	1.06 × 10^−1^	IGS	LSC; SSC
42	9996	R	18	9996	0	1.06 × 10^−1^	IGS	LSC
43	10,283	F	18	31,730	0	1.06 × 10^−1^	IGS	LSC
44	10,322	R	18	118,843	0	1.06 × 10^−1^	IGS	LSC; IRA
45	10,548	F	18	133,365	0	1.06 × 10^−1^	*ycf1*	LSC
46	31,728	F	18	125,951	0	1.06 × 10^−1^	IGS	LSC
47	39,812	F	18	40,698	0	1.06 × 10^−1^	*trnS* *-UGA*	LSC; SSC
48	40,022	R	18	69,093	0	1.06 × 10^−1^	IGS	LSC
49	40,700	F	18	123,827	0	1.06 × 10^−1^	IGS	LSC
50	43,446	F	18	45,670	0	1.06 × 10^−1^	*psaB*	SSC
51	40,022	R	18	69,093	0	1.06 × 10^−1^	IGS	LSC
52	40,700	F	18	123,827	0	1.06 × 10^−1^	IGS	LSC
53	43,446	F	18	45,670	0	1.06 × 10^−1^	*psaB*, *psaA*	LSC

F: forward; I: inverted; IGS: intergenic space.

**Table 6:** Simple sequence repeats (SSRs) in the *Q. acutissima* chloroplast genome.

ID	Repeat Motif	Length (bp)	Start	End	Region	Gene	ID	Repeat Motif	Length (bp)	Start	End	Region	Gene
1	(A)10	9	1809	1818	LSC		34	(T)10	9	55,713	55,722	LSC	
2	(C)14	13	4433	4446	LSC		35	(T)10	9	59,591	59,600	LSC	
3	(T)11	10	4697	4707	LSC		36	(T)10	9	60,063	60,072	LSC	
4	(A)10	9	4939	4948	LSC	*trnK-UUU*	37	(T)10	9	64,092	64,101	LSC	*accD*
5	(T)11	10	7001	7011	LSC		38	(A)11	10	64,266	64,276	LSC	
6	(T)10	9	7746	7755	LSC		39	(AT)7	13	64,570	64,583	LSC	
7	(A)10	9	8174	8183	LSC		40	(T)14	13	64,945	64,958	LSC	
8	(A)12	11	8590	8601	LSC	*psbK*	41	(T)13	12	66,170	66,182	LSC	
9	(A)11	10	8920	8930	LSC		42	(T)11	10	68,616	68,626	LSC	*petA*
10	(A)10	9	9465	9474	LSC		43	(T)11	10	70,730	70,740	LSC	
11	(A)10	9	10,161	10,170	LSC		44	(T)11	10	71,398	71,408	LSC	
12	(A)11	10	13,547	13,557	LSC		45	(T)11	10	73,389	73,399	LSC	
13	(T)12	11	15,345	15,356	LSC		46	(AT)6	11	77,274	77,285	LSC	*clpP*
14	(T)10	9	16,160	16,169	LSC		47	(TA)7	13	82,928	82,941	LSC	*petD*
15	(A)12	11	18,692	18,703	LSC	*rpoC2*	48	(A)11	10	85,781	85,791	LSC	
16	(T)12	11	21,295	21,306	LSC	*rpoC2*	49	(T)10	9	86,100	86,109	LSC	
17	(T)14	13	25,299	25,312	LSC		50	(T)10	9	88,820	88,829	LSC	
18	(T)10	9	28,563	28,572	LSC		51	(T)11	10	114,070	114,080	IRA	
19	(T)10	9	29,651	29,660	LSC		52	(T)12	11	118,582	118,593	SSC	
20	(T)11	10	30,275	30,285	LSC		53	(A)11	10	118,695	118,705	SSC	
21	(C)14	13	30,428	30,441	LSC		54	(T)11	10	119,000	119,010	SSC	
22	(T)11	10	31,731	31,741	LSC		55	(A)10	9	119,794	119,803	SSC	
23	(A)10	9	32,094	32,103	LSC		56	(T)11	10	122,199	122,209	SSC	*ndhD*
24	(A)10	9	33,986	33,995	LSC		57	(A)10	9	122,546	122,555	SSC	
25	(A)13	12	34,775	34,787	LSC		58	(AT)8	15	123,832	123,847	SSC	
26	(A)10	9	34,955	34,964	LSC		59	(T)11	10	125,812	125,822	SSC	
27	(A)10	9	36,485	36,494	LSC		60	(T)11	10	125,954	125,964	SSC	
28	(AT)6	11	39,819	39,830	LSC		61	(T)11	10	130,262	130,272	SSC	
29	(T)10	9	41,238	41,247	LSC	*trnfM-CAU*	62	(A)10	9	130,487	130,496	SSC	
30	(T)11	10	53,217	53,227	LSC		63	(T)10	9	133,465	133,474	SSC	*ycf1*
31	(A)10	9	53,726	53,735	LSC		64	(T)13	12	134,042	134,054	SSC	*ycf1*
32	(T)15	14	54,110	54,124	LSC		65	(A)11	10	137,468	137,478	SSC	
33	(A)11	10	54,990	55,000	LSC								

## Abbreviations

LSC	Large single copy
SSC	Small single copy
IR	Inverted repeat
Cp	Chloroplast
BI	Bayesian inference
A	Adenine
T	Thymine
G	Guanine
C	Cytosine

## Appendix A

**Table A1:** The number of genes in the *Q. acutissima* cp genome.

Region	Number of CDS	Number of tRNA	Number of rRNA	Total
LSC region	62	25	0	87
SSC region	13	1	0	14
IRA region	6	7	4	17
IRB region	7	7	4	18

**Table A2:** Codon-anticodon recognition patterns and codon usage of the *Q. acutissima* chloroplast genome.

Amino Acid	Codon	No.	RSCU	tRNA	Amino Acid	Codon	No.	RSCU	tRNA
Ala	GCG	164	0.47		Pro	CCA	313	1.13	*trnP* *-TGG*
Ala	GCC	224	0.64		Pro	CCC	226	0.82	
Ala	GCU	630	1.79		Pro	CCU	409	1.48	
Ala	GCA	388	1.1		Pro	CCG	161	0.58	
Cys	UGU	221	1.44		Gln	CAG	215	0.45	
Cys	UGC	86	0.56	*trnC* *-GCA*	Gln	CAA	731	1.55	*trnQ* *-TTG*
Asp	GAC	209	0.39	*trnD* *-GTC*	Arg	CGU	337	1.26	*trnR* *-ACG*
Asp	GAU	870	1.61		Arg	AGA	500	1.87	*trnR* *-TCT*
Glu	GAA	1064	1.5	*trnE* *-TTC*	Arg	CGA	358	1.34	
Glu	GAG	357	0.5		Arg	AGG	183	0.68	
Phe	UUU	983	1.3		Arg	CGG	118	0.44	
Phe	UUC	535	0.7	*trnF* *-GAA*	Arg	CGC	109	0.41	
Gly	GGU	580	1.27		Ser	AGC	125	0.37	*trnS* *-GCT*
Gly	GGG	330	0.72		Ser	UCU	557	1.66	
Gly	GGA	706	1.55		Ser	UCA	397	1.18	*trnS* *-TGA*
Gly	GGC	206	0.45	*trnG* *-GCC*	Ser	UCC	349	1.04	*trnS* *-GGA*
His	CAU	486	1.54		Ser	AGU	391	1.17	
His	CAC	145	0.46	*trnH* *-GTG*	Ser	UCG	193	0.58	
Ile	AUC	458	0.58		Thr	ACU	538	1.6	
Ile	AUA	758	0.97		Thr	ACG	160	0.48	
Ile	AUU	1139	1.45		Thr	ACC	247	0.73	*trnT* *-GGT*
Lys	AAG	379	0.5		Thr	ACA	402	1.19	*trnT* *-TGT*
Lys	AAA	1062	1.4		Val	GUU	508	1.41	
Leu	UUG	572	1.22	*trnL* *-CAA*	Val	GUC	181	0.5	*trnV* *-GAC*
Leu	UUA	894	1.9		Val	GUA	547	1.52	
Leu	CUU	583	1.24		Val	GUG	207	0.57	
Leu	CUA	373	0.79	*trnL* *-TAG*	Trp	UGG	462	1	*trnW* *-CCA*
Leu	CUC	204	0.43		Tyr	UAC	212	0.42	*trnY* *-GTA*
Leu	CUG	198	0.42		Tyr	UAU	792	1.58	
Met	AUG	620	1	*trnI* *-CAT*	Stop	UAA	47	1.6	
Asn	AAU	1004	1.5		Stop	UAG	22	0.75	
Asn	AAC	304	0.46		Stop	UGA	19	0.65	

RSCU: Relative Synonymous Codon Usage.
